# Effective Communications Techniques for Communities to Reach Older Adults in a Natural Disaster

**DOI:** 10.1093/geroni/igaa057.2426

**Published:** 2020-12-16

**Authors:** Patricia Fletcher

**Affiliations:** Federal Emergency Management Agency, Washington, District of Columbia, United States

## Abstract

Framing timely messages during an emergency is often underestimated but is vitally essential and necessary, especially for the older population. While there is existing research on disaster and older adults, there is a gap in the literature that focuses on a 21st-century communication model that reaches at-risk populations. This presentation explores how practitioners can inspire change in the delivery of an emergency message to communities. Appropriately designed health communications will provide extensive knowledge about the health crisis and improve public health literacy among older adults in disaster preparedness, response, and recovery, creating messages that influence necessary behavior change during the emergency. Additionally, recognizing how collaborative partnerships with Federal, Local, State government, trusted relief agencies, and community groups benefit from designing an organizational structure to disseminate information to the community. Part of a symposium sponsored by Disasters and Older Adults Interest Group.

